# Supplementary figures and images for: Unrestrained fatty acid oxidation triggers heart failure in mice via cardiolipin loss and mitochondrial dysfunction
Source: J Clin Invest. 2026 May 1;136(9):e202528. doi: 10.1172/JCI202528 (PMC13132376; doi:10.1172/JCI202528)

Figure S1D original Western Blot

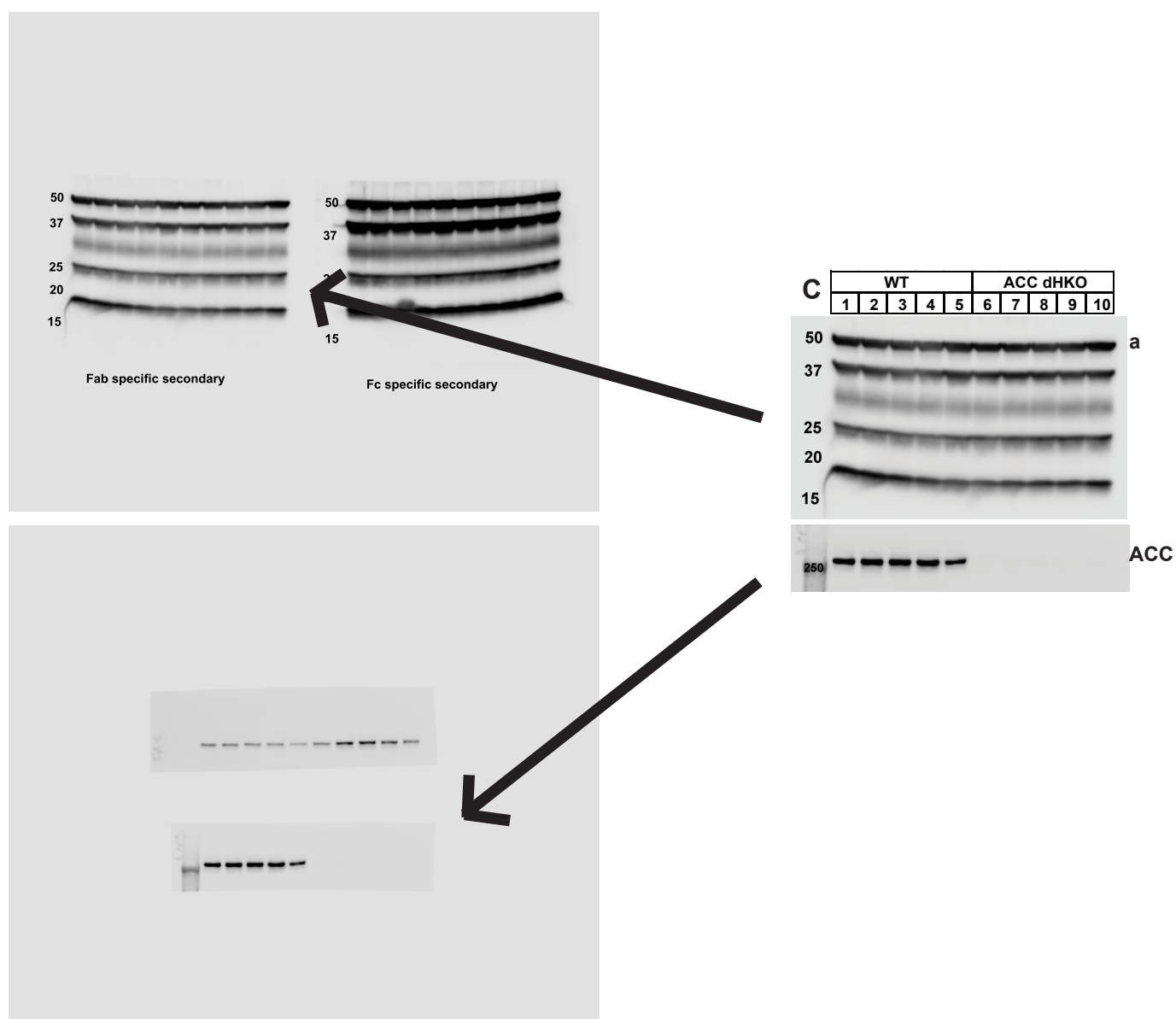

Figure S2K original Western Blot

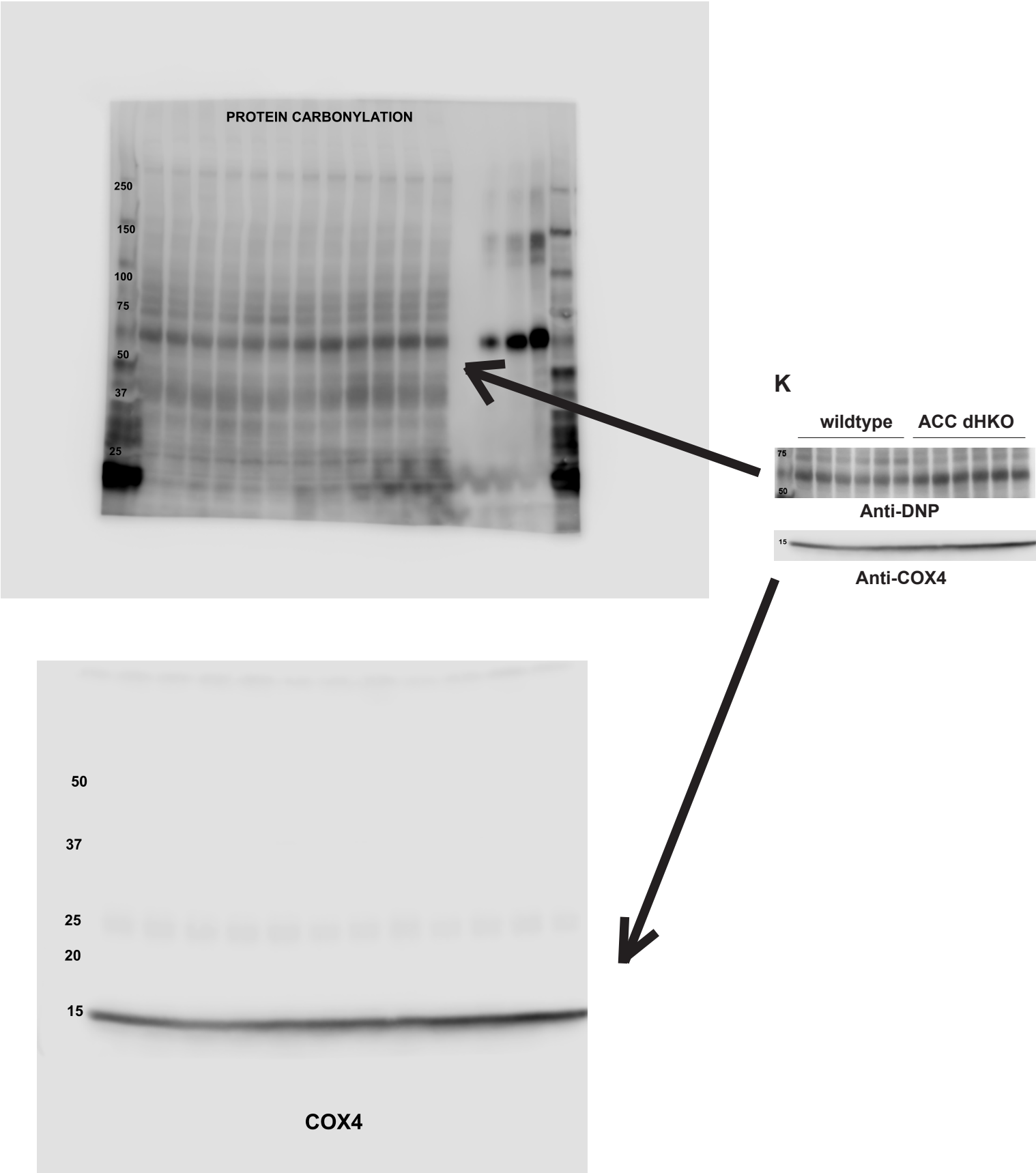

Supplement: Unedited blot and gel images [file jci-136-202528-s085.pdf]
